# Studies in rats of a target specific and reversible general anesthetic with a favorable safety profile

**DOI:** 10.1371/journal.pone.0335589

**Published:** 2025-11-04

**Authors:** Zheng Xie, Robert Fong, Aaron P. Fox

**Affiliations:** 1 Department of Anesthesia and Critical Care, The University of Chicago, Chicago, Illinois, United States of America; 2 Department of Pharmacological and Physiological Sciences, The University of Chicago, Chicago, Illinois, United States of America; University of Split Faculty of Medicine: Sveuciliste u Splitu Medicinski fakultet, CROATIA

## Abstract

Delirium and cognitive decline are linked to clinically relevant anesthetics in the vulnerable elderly population, prompting the need for new and safer anesthetic strategies. Most general anesthetics potentiate the activity of GABA_A_ receptors. However, these drugs act on myriad other targets, causing unwanted effects. Dexmedetomidine (Dex), a selective α_2_ adrenergic receptor agonist, is associated with reduced incidences of delirium and cognitive decline in the elderly. Unfortunately, despite its sedative effect, Dex is not suitable for general anesthesia when used alone. We previously demonstrated that enhancing Dex with low doses of either sevoflurane or propofol resulted in a potent general anesthetic that was rapidly reversible. In this study we assessed whether Dex enhanced by magnesium (Mg^2+^) infusion could produce a general anesthetic. Mg^2+^ is an essential ion in the body, possessing sedative effects attributable to antagonizing NMDA receptors and voltage-gated Ca^2+^ channels and it may indirectly potentiate GABAergic signaling. Mg^2+^ has been shown to be neuroprotective and safe to use even in pregnant women. Mg^2+^ is a safer adjunct agent than either sevoflurane or propofol. For this study, rats of both sexes were anesthetized with a combination of Dex and Mg^2+^ and then underwent procedures to determine the efficacy of the anesthetic. Dex with Mg^2+^ produced an effective general anesthetic that was reversed by a combination of low dose atipamezole, an α_2_ competitive antagonist, and caffeine. We compared Dex supplemented with Mg^2+^ to Dex supplemented with midazolam, a selective positive GABA_A_ modulator and found that immobility, antinociception, EEG signatures, and hemodynamic profiles were comparable. Our findings showed that activation of α_2_ receptors by Dex, with blockade of NMDA receptors/ Ca^2+^ channels by Mg^2+^ produce an effective and reversible general anesthetic with possible neuroprotective properties that may be appropriate for cognitively vulnerable patients like the elderly.

## Introduction

Though modern anesthesia is generally considered benign, the elderly constitute a vulnerable population who will benefit from safer anesthetics. Current anesthetics, like sevoflurane and propofol, were developed decades ago [1,2]. In the vulnerable elderly population, these anesthetics are linked with delirium and cognitive dysfunction [3]. The very young likely represent another vulnerable population as increased neuroapoptosis and cognitive changes have been observed in neonates of a variety of animal species including non-human primates [4–6]. Even though unambiguous evidence of neurotoxicity in children remains to be demonstrated, the search for new and potentially safer anesthetic regimens represents a valuable endeavor.

To be useful, an anesthetic must exhibit four characteristics; amnesia, unconsciousness, antinociception and immobility [7]. While amnesia is difficult to evaluate in animal models, our current study is able to characterize unconsciousness, antinociception and immobility.

Dexmedetomidine (Dex) is thought to possess a favorable safety profile since elderly human patients exhibit fewer incidences of delirium and cognitive decline [8–10] and in young animals it is thought to be neuroprotective [11,12]. By itself Dex is not an effective anesthetic: Dex alone did not suppress responses to noxious stimuli in rats [13]. In a previous study we showed that Dex, supplemented with low subanesthetic doses of two common anesthetics, propofol or sevoflurane, produced an efficacious general anesthetic that was appropriate for surgery [13]. Although this strategy employed low doses of propofol and sevoflurane, it is unclear whether the decreased dose of these agents would mitigate their adverse neurocognitive effects in the elderly [3,14,15]. An alternative approach is to eliminate these agents entirely and to replace them with another neuroprotective agent. To this end we evaluated whether Dex supplemented with magnesium sulfate (Mg^2+^) produced a complete anesthetic, as Mg^2+^ has been shown to be neuroprotective in a variety of studies [16–18], since it regulates Ca^2+^ homeostasis by blocking voltage-gated Ca^2+^ channels, tamps down on neuroinflammation, and protects against excitotoxicity (also via a Ca^2+^-dependent mechanism) [19–21]. Rats are physiologically different than humans. Even so, the Mg^2+^ dosing regimen was initially based on human levels used for premature labor or preeclampsia during pregnancy [22]. We compared Dex supplemented with Mg^2+^ to Dex supplemented with midazolam, a drug that selectively potentiates GABA_A_ receptor currents [23]. Midazolam is more target selective than either propofol or volatile anesthetics and thus will have fewer pleiotropic effects. Notably, midazolam is a strongly amnestic agent even at the modest concentrations employed in this study [23]. We observed that low-dose midazolam combined with Dex created a potent general anesthetic.

Previously, we found that we could reverse Dex with a combination of a low dose of atipamezole, an α_2_adrenergic receptor competitive antagonist, and caffeine [24]. This reversal strategy was tested in the current study.

For these studies 3 groups (n = 8) of female rats (24 in total) and 2 groups (n = 8) of male rats (16 rats total), were tested. The same rats were studied at different ages. As anesthetic requirements vary with age, female and male rats were tested at 3–4 months (“young”), 11–12 months (“older”) and 18–19 months (“old male rats”) or 20–21 months of age (“old female rats”).

## Materials and methods

### Ethics and animals

The University of Chicago Institutional Animal Care and Use Committees (protocol #42437) approved this animal study. Rats were cared for by veterinary staff. We used 3 groups (8 per group) of female rats and 2 groups (8 per group) male adult Sprague Dawley rats (Charles River, Wilmington, MA). We initially tested rats at 3–4 months of age. Two groups of each sex were kept until they were 11–12-month-old. The same rats were tested at different ages. Female rats weighed 250–436 g at 3–4 months of age, while males were 275–468 g. There was no difference in the mean weights at 3–4 months 340.5 g vs 366.3 g, p = 0.16, unpaired t-test. At 12 months, females weighed 430–630 g and males were 412–580 g. There was no difference in the mean weights at 12 months 520.5 g vs 522.6 g, p = 0.9, unpaired t-test. Eight female rats aged 11–12-month-old were used for surgery. Six females were kept to the age of 20–21 months (310–390 g) while four males aged 18–19 months weighed from 455 to 562 g. Not every experiment was repeated in the male rats. Their primary purpose was to test whether data collected in the female rats was reproducible in male rats. As the rats grew older, the animals died or developed lesions/ tumors or had other health issues that required euthanasia. Thus, we were left with 6 female and 4 male surviving animals. As they aged, male rats died more frequently than did female rats. The oldest male rats were not perfectly age matched with the female rats, the males were 2 months younger, since a minimum of 4 rats were required for the study. It was decided to test the male rats at an earlier age to guarantee a group size of 4. Because both surgery and the tail clamp represented noxious stimuli, and thus report similar data, only one group of female rats was sacrificed to surgery when they were 11–12 months of age. Because of their fragility, the oldest rats were anesthetized a single time.

The oldest female rats lost weight compared to those of 11–12-month-old. The oldest male rats maintained the same weight of those of 11–12-month-old. Otherwise, experimental conditions were identical to those previously described ^20^. Rats served as their own controls. Heart rate, respiratory rate, blood pressure and blood O_2_ saturation were monitored throughout each anesthesia sessions, using a Kent Scientific Physiosuite or a Kent Scientific Coda System (for BP). The Coda BP system was purchased after the experiments on the youngest age group were already completed. Each rat was anesthetized no more than 4 times during each age period of 3–4 months or 11–12 months. During the age period of 18–21 months, each rat was anesthetized one time due to their fragility. At the conclusions of the study, rats were euthanized by the animal facility staffers using CO_2_ overdose, followed by decapitation. In experiments where surgery was performed, the rats were sacrificed, after the wound were closed with sutures, by an overdose of propofol (20 mg/kg) and decapitation by veterinary staff.

**Calibrated Noxious Stimulus:** A calibrated tail clamp, [13], was used to assess anesthetic efficacy. The stimulus was generated with Kelly forceps that were used to clamp each rat’s tail where it was exactly 5 mm in diameter. Clamping the tail to the first stop on the forceps for 30 seconds, or until a response was evoked, provided a consistently reproducible stimulus.

**Abdominal surgery:** To assess whether Dex in combination with Mg^2+^ could provide robust general anesthesia, we performed abdominal surgery involving incision of the skin, underlying abdominal muscles and peritoneum. Fig 8A shows a rat after surgery. Immobility during surgery represents the gold standard for successful anesthesia [7].

**Fig 1:**
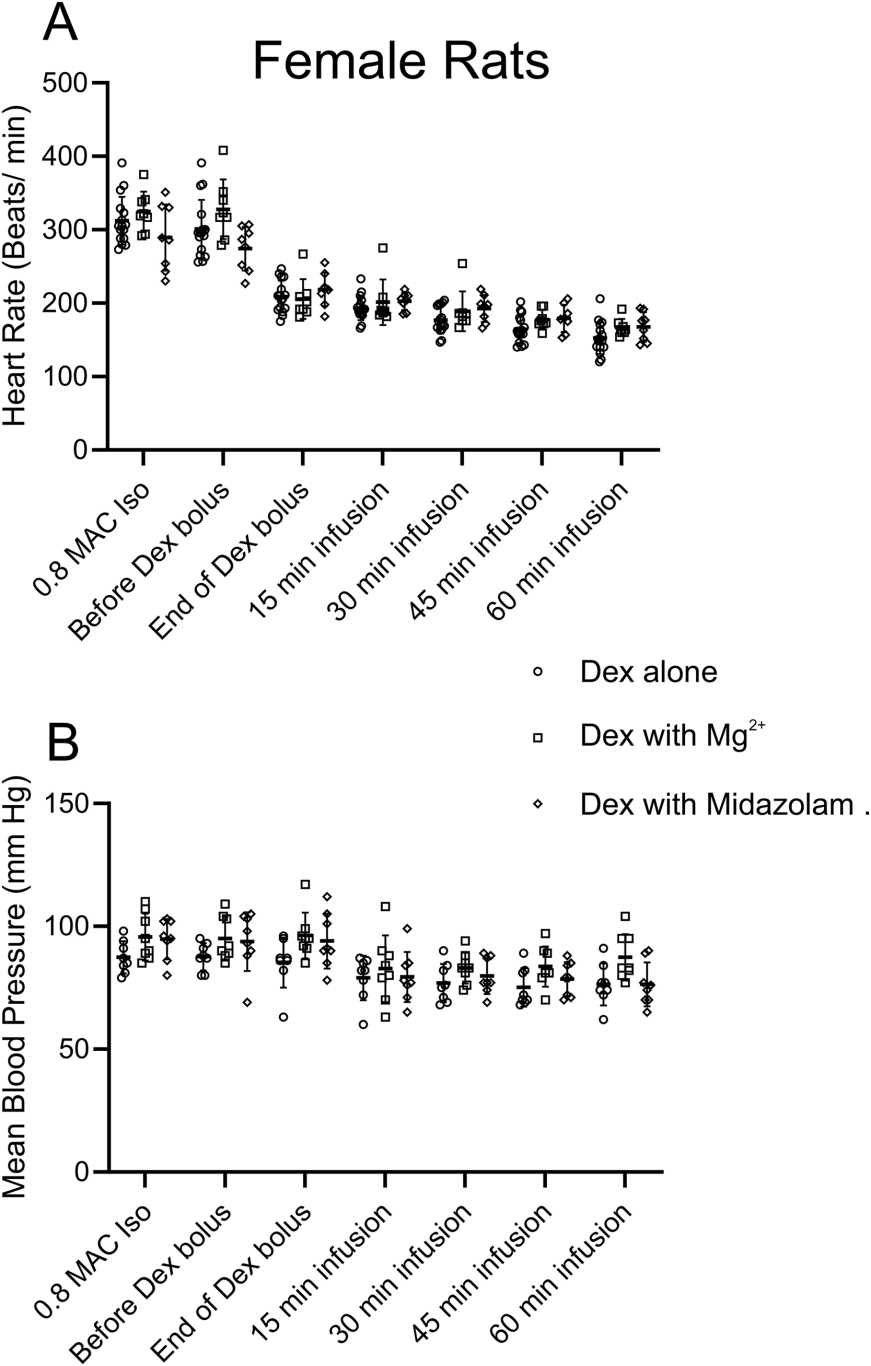
Dex alone or Dex with Mg^2+^ or Dex with Midazolam produced a similar reduction in HR without changing BP in older female rats (11–12-months-old). **A**, comparisons of HR at different times in the experiment for Dex alone (applied as 10 µg/kg bolus, followed by 12 µg/kg/hr infusion), Dex with Mg^2+^ (applied as a 50 mg/kg bolus, followed by 25 mg/kg/hr infusion) and Dex with Midazolam (applied as a 0.6 mg/kg bolus, followed by 0.3 mg/kg/hr infusion). In all three cases the Dex application was identical. For this analysis a repeated measures two-way ANOVA with Tukey’s multiple comparisons test was employed (n = 8, adjusted p value): Dex with Mg^2+^: HR taken just before the Dex bolus under Isoflurane was compared to the rest of the time points, Before Dex bolus vs. End of Bolus, p < 0.001: Before Dex bolus vs. t = 15, p < 0.001: Before Dex bolus vs. t = 30, p < 0.001: Before Dex bolus vs. t = 45, p < 0.001: Before Dex bolus vs. t = 60, p < 0.001. Dex with Midazolam: We compared the HR taking before Dex bolus under Isoflurane to the rest of the time points, Before Dex bolus vs. End of Bolus, p < 0.05: Before Dex bolus vs. t = 15, p < 0.0001: Before Dex bolus vs. t = 30, p < 0.0001: Before Dex bolus vs. t = 45, p < 0.0001: Before Dex bolus vs. t = 60, p < 0.0001. **B,** comparisons of mean blood pressure (MBP) at different time points showed minimal changes in these older female rats. There was no significant difference in MBP for any time points.

**Fig 2:**
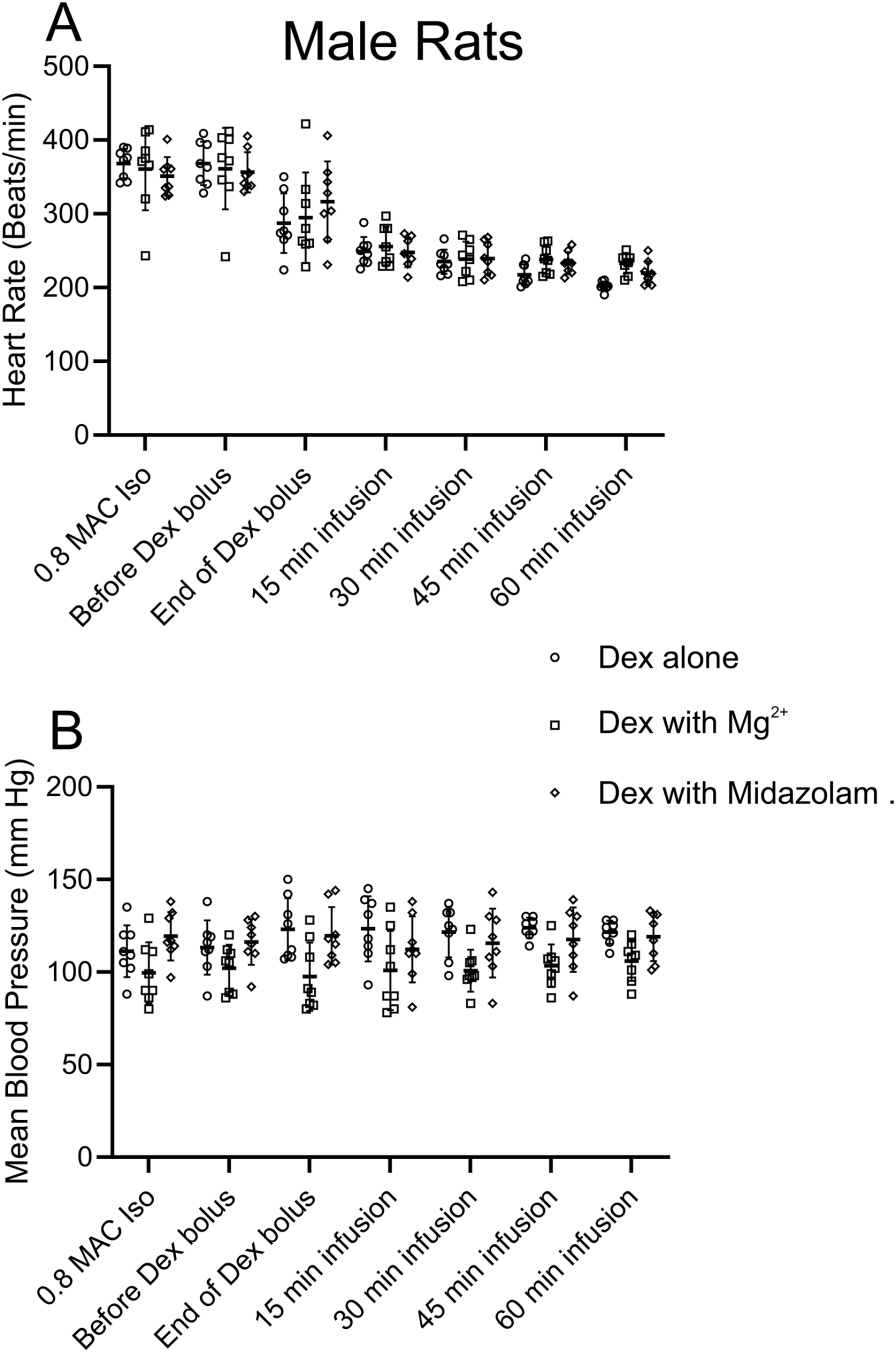
Dex alone or Dex with Mg^2+^ or Dex with Midazolam produced a similar reduction in HR without changing BP in older male rats (11–12-months-old). **A,** Comparisons of HR at different times for Dex at 10 µg/kg bolus, followed by 12 µg/kg/hr, Dex with Mg^2+^ at 50 mg/kg bolus, followed by 25 mg/kg/hr and Dex with Midazolam at 0.6 mg/kg bolus, followed by 0.3 mg/kg/hr. For this analysis a repeated measures two-way ANOVA with Tukey’s multiple comparisons test was employed (n = 8, adjusted p value): Dex with Mg^2+^: We compared the HR taking before Dex bolus under Isoflurane to the rest of the time points, Before Dex bolus vs. End of Bolus, p < 0.01: Before Dex bolus vs. t = 15, p < 0.001: Before Dex bolus vs. t = 30, p < 0.001: Before Dex bolus vs. t = 45, p < 0.001: Before Dex bolus vs. t = 60, p < 0.001. Dex with Midazolam: HR was compared before the Dex bolus under Isoflurane to the rest of the time points. Before Dex bolus vs. End of Bolus, p < 0.05: Before Dex bolus vs. t = 15, p < 0.0001: Before Dex bolus vs. t = 30, p < 0.0001: Before Dex bolus vs. t = 45, p < 0.0001: Before Dex bolus vs. t = 60, p < 0.0001. **B,** comparisons of mean blood pressure (MBP) at different time points showed minimal changes in these older female rats. There was no significant difference in MBP for any time points.

**Fig 3:**
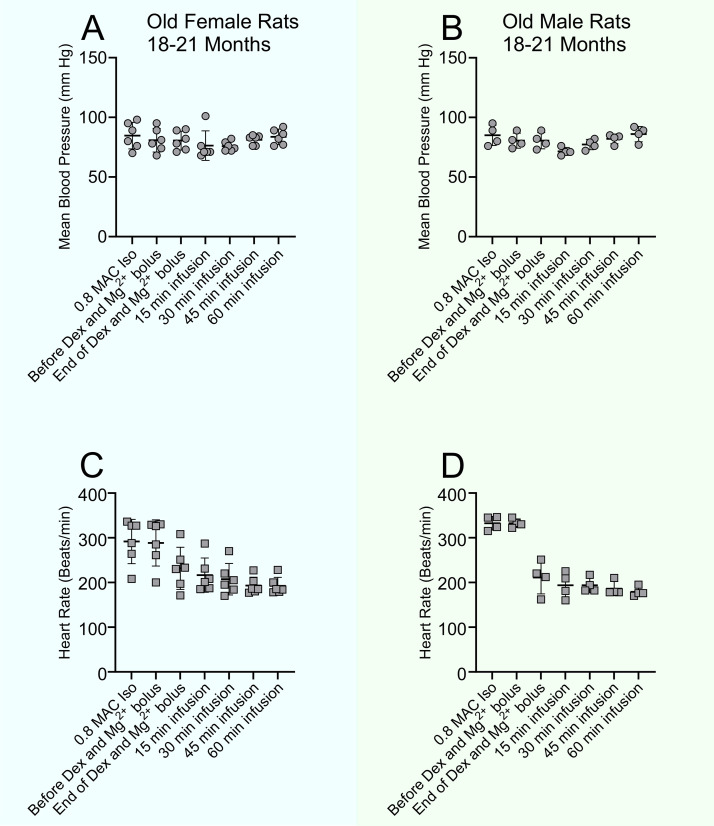
Vital signs before and during Dex with Mg^2+^ infusion in old rats, aged 18-21 months. Vital signs during Isoflurane 0.8 MAC and during Dex/Mg^2+^ infusion (Dex 10 µg/kg, Mg^2+^ 100 mg/kg bolus, followed by Dex 12 µg/kg/hr and Mg^2+^ 50 mg/kg/hr infusion) in oldest female (n = 6) (**A & C**) and male (n = 4) (**B & D**) rats at the age of 20-21 months and 18-19 months, respectively. Repeated measures one-way ANOVA with Geisser-greenhouse correction and Dunnett’s multiple comparison test were performed. While Dex/Mg^2+^ infusion caused a significant drop in HR, it produced insignificant changes in mean BP between the pre and post Dex and Mg^2+^ infusion mean BPs in both old female and male rats. The weights of the aged male rats (524 ± 48 g, n = 4) were like those when they were 11-12 months old (543 ± 31 g, n = 4, p = 0.52). Aged female rats (344 ± 29 g) were lower in weight compared to the same rats at 11-12 months (484 ± 35 g, n = 6, p < 0.0001). The weight of the female and male rats in these groups at 3-4 months old were 270 ± 18 g and 339 ± 36 g, respectively (n = 8, p < 0.001).

**Fig 4:**
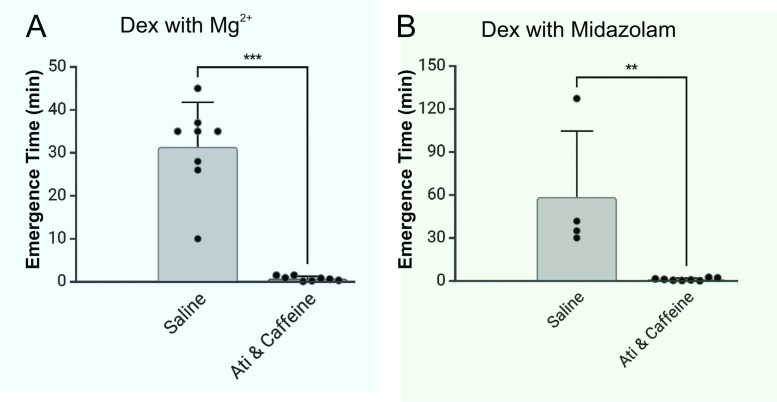
Emergence times for Dex supplemented with Mg^2+^ or Midazolam were dramatically accelerated by a reversal cocktail composed of low dose atipamezole and caffeine. **A,** Low dose atipamezole (Ati, 10 µg/kg) and caffeine (25 mg/kg) were assessed in a group of 8 female rats, aged 11-12 month. Each rat was exposed to Dex with Mg^2+^ in two separate sessions. Dex 10 µg/kg and Mg^2+^ 75 mg/kg were applied as boluses over 5 minutes via a pump, followed by Dex 12 µg/kg/hr and Mg^2+^ 37.5 mg/kg/hr infusion for 60 min. The rats received saline (control) at the end of one session and Ati & caffeine at the end of the other session. Rats recovered their righting reflex in 31.4 ± 10.40 min in the saline session and in 1.2 ± 0.9 min in the session where they received Ati & Caffeine (n = 8; p < 0.0001, paired t-test). **B,** Emergence times for Dex supplemented with midazolam were dramatically accelerated by a reversal cocktail composed of low dose Ati & Caffeine. Low dose Ati & caffeine were assessed in a different group of female rats, 11-12 month of age. Each rat was exposed to Dex with midazolam in two separate sessions. Dex 10 µg/kg and 0.6 mg/kg Midazolam were applied as a bolus over 5 minutes, followed by Dex 12 µg/kg/hr and Midazolam 0.3 mg/kg/hr infusion over 60 min. Saline (control) was administered in one session and Ati (10 µg/kg) & Caffeine (25 mg/kg) at the end of the other session. Rats recovered their righting reflex in 58.5 ± 46.11 min (n = 4) in the saline session and in 1.1 ± 0.87 min in the Ati & Caffeine session (n = 8; p < 0.01, unpaired t-test). Plotted is the mean value with SD.

**Fig 5:**
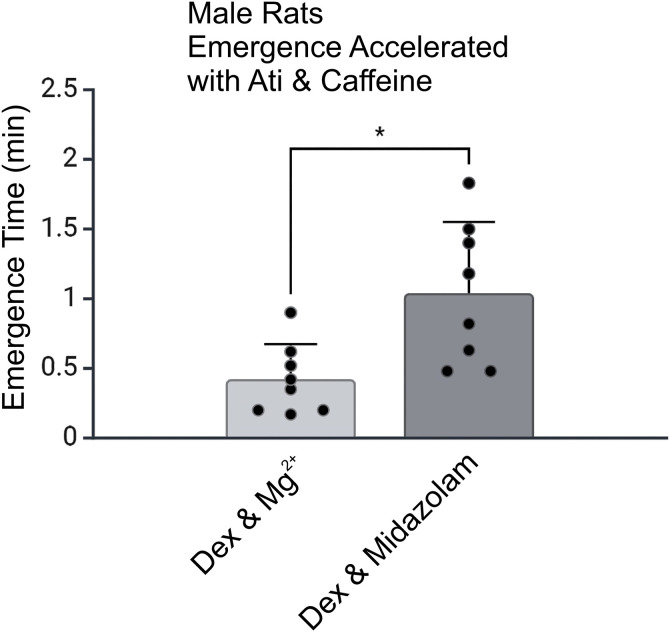
Emergence times with a reversal cocktail of low dose atipamezole and caffeine, in older male rats. In a group of 8 male rats, 12 months old, the efficacy of atipamezole (10 µg/kg) and caffeine (25 mg/kg) was assessed. Rats received Dex 10 µg/kg and Mg^2+^ 100 mg/kg bolus over 5 minutes, followed by Dex 12 µg/kg/hr and Mg^2+^ 50 mg/kg/hr infusion over one hour. At the end of the infusion, they were injected with Ati & caffeine. In a different session, rats received Dex 10 µg/kg and Midazolam 0.6 mg/kg bolus over 5 minutes, followed by Dex12 µg/kg/hr and Midazolam 0.3 mg/kg/hr infusion over one hour. Atipamezole and caffeine was injected at the end. Rats recovered their righting reflex in 0.4 ± 0.3 min in Dex & Mg^2+^ session and in 1.0 ± 0.5 min in Dex & Midazolam session (n = 8) (p < 0.03, paired t test). Plotted is the mean value with SD.

**Fig 6:**
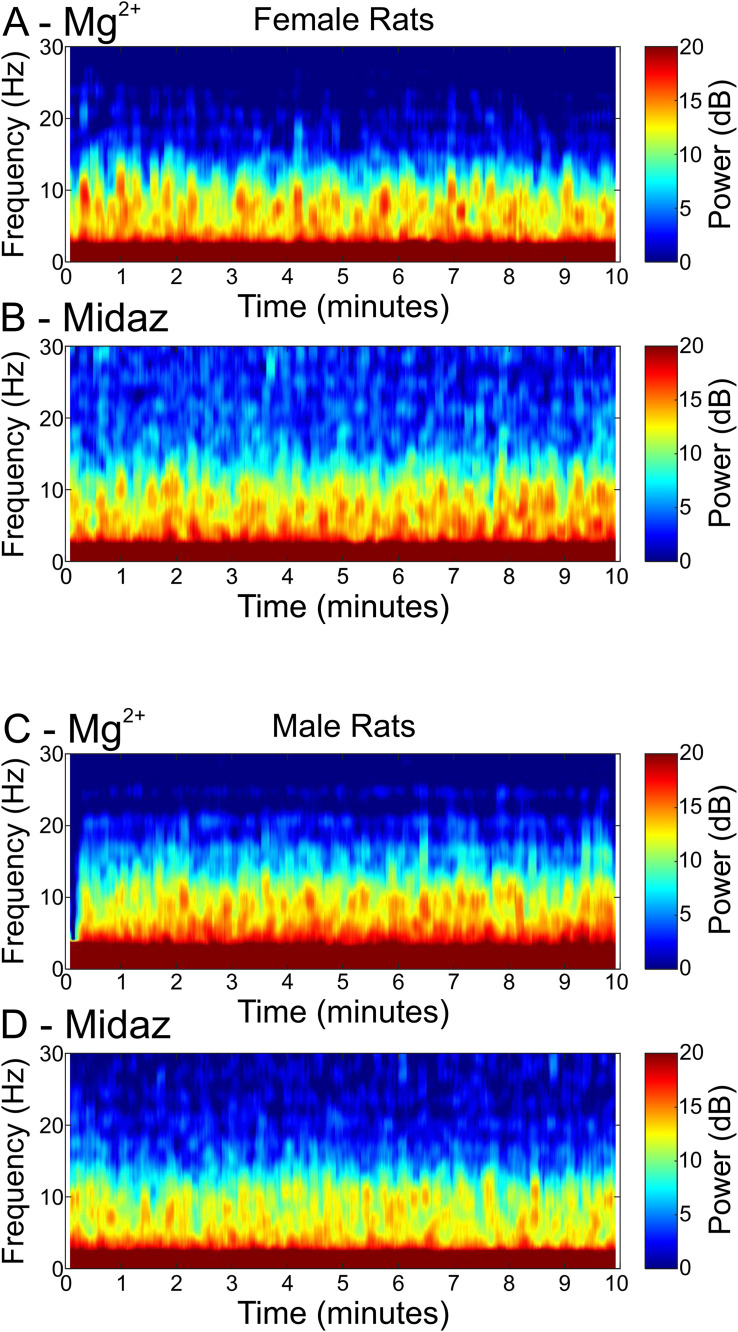
Similar EEG spectrograms obtained in a female rat (A and B) and a male rat (C and D) exposed to either Dex supplemented with Mg^2 +^ (A, C) or Dex supplemented with Midazolam (B, D). A and B show data obtained from recordings obtained from the anterior lead of a 12-month-old female rat. The rat received two anesthesia sessions. Top and bottom traces represent 10-minute spectrograms recorded under Dex (10 µg/kg, followed by 12 µg/kg/hr infusion) supplemented with Mg^2+^ (50 mg/kg bolus, followed by 25 mg/kg/hr infusion) or another 10-minute spectrogram recorded under Dex (10 µg/kg, followed by 12 µg/kg/hr infusion) supplemented with Midazolam (0.6 mg/kg bolus, followed by 0.3 mg/kg/hr infusion), respectively. C and D show recordings obtained from the anterior lead of a 12-month-old male rat. The rat received two anesthesia sessions. Top and bottom traces represent 10-minute spectrograms recorded under Dex (10 µg/kg, followed by 12 µg/kg/hr infusion) supplemented with Mg^2+^ (100 mg/kg bolus, followed by 50 mg/kg/hr infusion) and another 10-minute spectrogram recorded under Dex (10 µg/kg, followed by 12 µg/kg/hr infusion) supplemented with Midazolam (0.6 mg/kg bolus, followed by 0.3 mg/kg/hr infusion), respectively.

**Fig 7:**
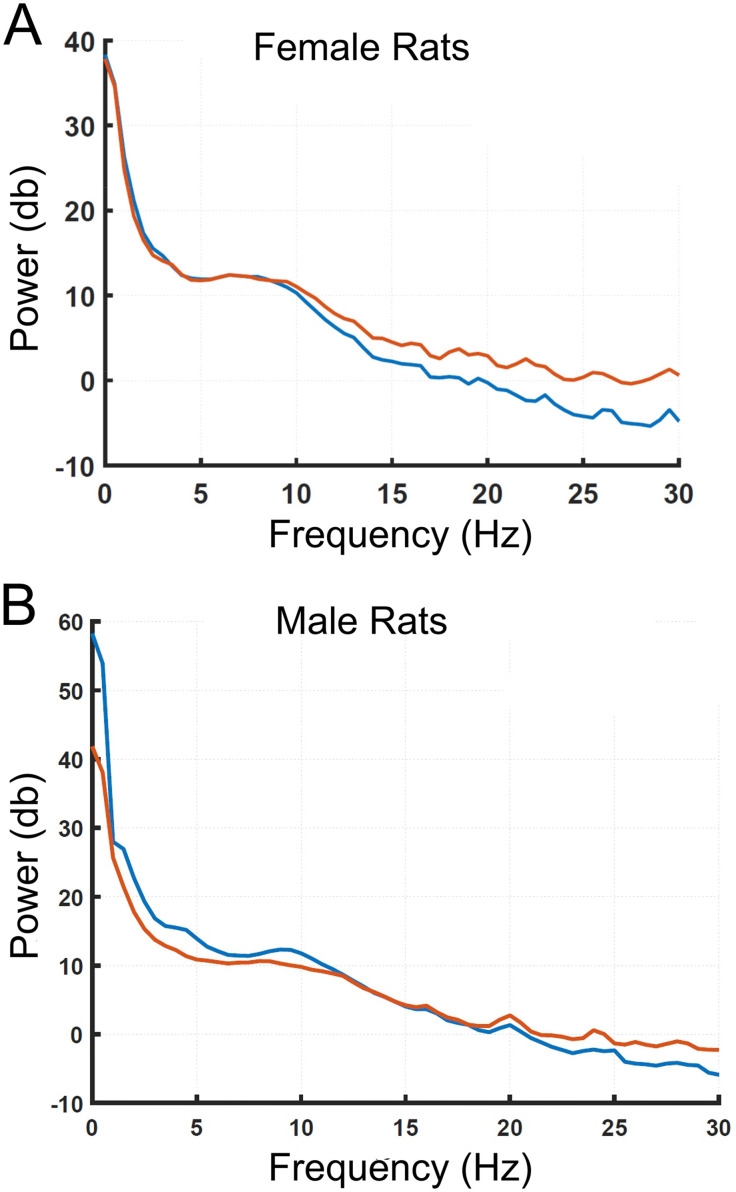
Similar power spectra were observed in rats anesthetized with Dex supplemented with Mg^2+^ or with Dex supplemented with midazolam. **A,** shows EEG data from a 5-minute epoch averaged from 8 female rats (11–12-month-old) in two separate sessions. In one session the rats received Dex with Mg^2+^ (blue) and in the other session, Dex with Midazolam (Red). The order of the drugs was randomized. Dex (10 µg/kg bolus, followed by 12 µg/kg/hr infusion) with Mg^2+^ (50 mg/kg bolus, followed by 25 mg/kg/hr infusion) and under Dex (10 µg/kg, followed by 12 µg/kg/hr infusion) with Midazolam (0.6 mg/kg bolus, followed by 0.3 mg/kg/hr infusion). Power (dB) frequency bands between Dex with Mg^2+^ and Dex with Midazolam are shown as delta (0.5-4 Hz) p = 0.85, 19.47 ± 7.71 vs 18.76 ± 7.52; theta (4-8 Hz) p = 0.61, 12.17 ± 0.19 vs 12.11 ± 027; alpha (8-12 Hz) p = 0.41, 9.75 ± 2.13 vs 10.52 ± 1.48; spindle (12-15 Hz) p < 0.05* 4.03 ± 1.63 vs 6.09 ± 1.32; and beta (15-25 Hz) p = 0.001 ***, −0.77 ± 1.97 vs 2.45 ± 1.39, n = 8). Note that Delta, theta and alpha frequency bands comprise >95% of the full frequency band power. These low frequency bands were not significantly different between two Dex based combinations. For both anesthetic conditions, delta bands dominated. The beta frequency band was significant higher in Dex with midazolam than in Dex with Mg^2+^. The results suggested that the depth of anesthesia based on EEG between the two anesthesia conditions was similar or even slightly deeper under Dex with Mg^2+^. Both spectrograms and power spectra were obtained near the 30-minute point of the infusion. An unpaired-T test was used to compare the frequency bands between two anesthesia conditions. **B,** shows EEG data from a 5-minute epoch averaged from 6 male rats (11–12-month-old) in two separate sessions. In one session the rats received Dex with Mg^2+^ (blue) and in the other session, Dex with Midazolam (Red). The anesthesia conditions were identical to those described for the female rats in part **A.** Power (dB) frequency bands between Dex with Mg^2+^ and Dex with Midazolam are shown as delta (0.5-4 Hz) p = 0.36, 24.86 ± 12.70 vs 19.64 ± 8.75; theta (4-8 Hz) p < 0.05*, 12.83 ± 1.63 vs 10.83 ± 0.61; alpha (8-12 Hz) p = 0.06, 11.06 ± 1.32 vs 9.70 ± 0.8; spindle (12-15 Hz) p = .97 6.23 ± 1.66 vs 6.20 ± 1.53; and beta (15-25 Hz) p = 0.36: 0.24 ± 2.27 vs 1.35 ± 1.68, n = 6). Note that under both anesthetic conditions, delta bands were the dominant power and not different between the two periods. Delta, theta and alpha frequency bands comprised >95% of the full frequency power under both conditions and these low frequency bands are not significantly different between two Dex based combinations. The data suggest that the depth of anesthesia based on EEG was similar between the two anesthesia sessions. Both spectrograms and power spectra were obtained near the 30-minute point of the infusion. The unpaired-T test was used to compare the frequency bands between two anesthesia conditions.

**Fig 8:**
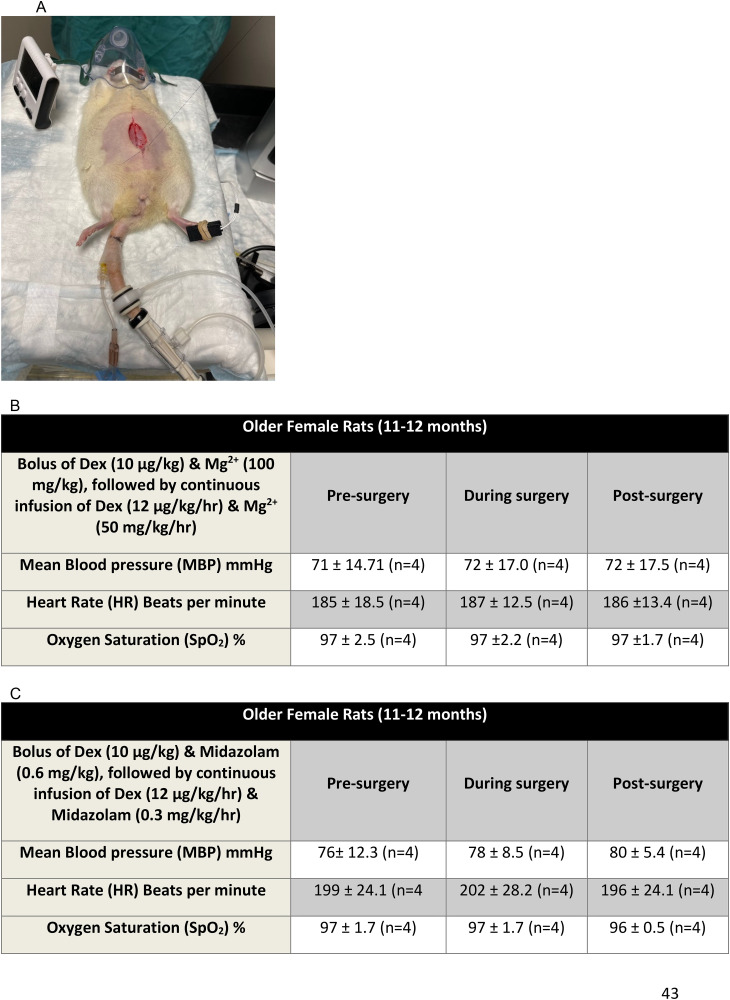
A representative illustration of an older female rat (11-12 months) that underwent abdominal surgery that received Dex supplemented with Mg^2+^ with no other agents. Vital signs were unchanged when surgery was performed with either Dex/Mg^2+^ or Dex/Midazolam. **A,** The rat received a bolus of Dex (10 µg/kg) and a bolus of Mg^2+^ (100 mg/kg) across 5 minutes, followed by an infusion of Dex (12 µg/kg/hr) and Mg^2+^ (50 mg/kg/hr infusion). The rat showed no response to the surgery. Surgery was performed at 15 min after the bolus of Dex and Mg^2+^ was complete and finished at ~30 min into the continuous infusion of Dex/Mg^2+^ infusion. No changes in vital signs were observed during the surgery. **B,** Vital signs obtained from 4 female rats (11-12 months) before, during and after surgery using Dex supplemented with Mg^2+^. Surgery was performed 15 min after the bolus of Dex and Mg^2+^ and finished ~30 min into the Dex/Mg^2+^ infusion. **C,** Vital signs obtained from 4 female rats (11-12 months) before, during and after surgery using Dex supplemented with midazolam. Surgery was performed 15 min after the bolus of Dex and midazolam and finished ~30 min into the Dex/midazolam infusion. Note that there were no significant differences in vital signs, using a RM-ANOVA, before, during and after the surgery.

What does no change to a noxious stimulus mean? For blood pressure there was no change >5 mm Hg. Similarly for heart rate any change greater than 10 beats per minute that lasted for more than 10 seconds would be assessed as a response. Any change in respiratory rate of any magnitude was that lasted for more than 20 seconds was a response. These parameters were chosen to eliminate random fluctuations in BP, HR and RR that took place without stimulation.

**Measurements of responses:** The loss and the recovery of righting reflex (LORR and RORR) were used as the proxies for the loss and recovery of unconsciousness, respectively. The body movement in responding to the tail clamp was used to measure immobility. The changes in heart rate and blood pressure during surgery were used to measure nociception.

**Drugs:** Caffeine (Sigma-Aldrich, St Louis, part # C0750-5G, Lot#SLBD0505V) was administered as a bolus infusion via an IV line at the concentration described in the manuscript (see ref 20).

Atipamezole (also called Antisedan, Zoetis Pharmaceuticals, Parsip-pany, NJ, #RXANTISEDAN-10), an α_2_ adrenergic competitive antagonist, was administered as a bolus infusion via an IV line (see ref 20). The dosages varied as described in the manuscript.

Dexmedetomidine was administered via an infusion pump into the IV line (see ref 20).

Magnesium Sulfate (40 mg/ml in water), delivered into an IV line via a pump, was purchased from Hospira (NDC-0409-6729-03).

Midazolam (1 mg/ml), delivered into an IV line via a pump was purchased from Naco Healthcare, Fort Atkinson, WI.

**Dosing levels:** Rats have different dosing levels than humans, making direct comparisons difficult. Dexmedetomidine, applied via pump as a 10 mg/kg bolus over five minutes, followed by 12 mcg/kg/hr was used as the main agent in combination of either Mg^2+^ or a low dose of midazolam. Dex at this dose caused the rats to lose their righting reflex (LORR) but did not produce sufficient antinociception for tail clamp test or surgery [13].

In humans, a 4–6 g loading dose of Mg^2+^ is recommended, followed by 1–2 g/hr maintenance. Converting these numbers for a 70 kg patient, Mg^2+^ would be given at 60–85 mg/kg bolus and ~30 mg/kg/hr. At this dosing regimen, rats do not lose their righting reflex. We started with a 50 mg/kg bolus and 25 mg/kg/hr infusion of Mg^2+^ to supplement Dex. Mg^2+^ dose was increased by 50% in the next session only if rats responded to the noxious stimulus. The highest dose of Mg^2+^ we used in this study was a 100 mg/kg bolus and 50 mg/kg/hr infusion.

The midazolam dosing regimen used in the current study did not cause loss of righting in the rats.

**Sedation/ anesthesia:** Rats were placed in a gas-tight anesthesia chamber where they were exposed to 2% isoflurane (ref [24]). After rats became unconscious, they were removed from the anesthesia chamber and put on a nose cone where they received 1.8% isoflurane/O_2_/Air. A 24G intravenous (IV) catheter was inserted into a tail vein of each rat. In some rats, scalp EEG electrodes were inserted under anesthesia. EEG recordings were carried out in one group of each sex at age 11–12 months, since results were so consistent. Supplementary Figure 1 shows the EEG electrode placement. This procedure required extra anesthesia. The group size was sufficient for the power spectrum analysis. Isoflurane was terminated after the bolus dose of Dex or Dex with either Mg^2+^ or with midazolam was delivered. The tail clamp stimulus was applied only after the 15 minute-washout of isoflurane with 2 L O_2_/Air was complete.

**Dex infusion with and without a second agent:** Dex was administered via an infusion pump attached to the IV line (see ref [13]). Using a Y shaped microcatheter (Baxter Healthcare Corp, Deerfield, IL) allowed two drugs to be administered, Dex and Mg^2+^, without mixing until they reached the small IV catheter so changing the infusion rate of one drug did not affect delivery of the other. When the infusions were stopped, we disconnected the Y-type microcatheter from the end of the IV catheter. Reversal agents (atipamezole with caffeine) or saline were injected by syringe (ref [24]).

**Electroencephalogram (EEG) recording:** Scalp electrodes were used in both female and male rats. Two scalp electrodes [Astro-Med/ Grass Technologies] were placed by using a line between the anterior edge of both ears, between Bregma and Lambda as described in reference [25]. For EEG studies, rats were used at 11–12 months of age. One group of each sex were for EEG. The female group still had all 8 of its original members, while the male group was down to 6.

**Power Spectra:** Two types of power spectra were computed: conventional power spectra using the SYNAMP EDIT module, and spectrograms using MATLAB R2021, and the EEGLAB program, using time resolution of 10 seconds with 99% overlap. Details are provided in ref [13]. Power spectra (dB) were computed over 5-minute-long epoch of EEG, partitioned into 512-point epochs, and averaged, yielding a temporal resolution of 2 Hz. Power was calculated as the fraction of a specific frequency power, including delta (0.5–4 Hz), theta (4–8 Hz), alpha (8–12 Hz), spindle (12–15 Hz) and beta (15–25 Hz) by MATLAB R2021. The average powers in a group of 8 female rats in each frequency band were obtained and compared in one session during the infusion of Dex with Mg^2+^ to another session during the infusion of Dex with midazolam. Similar EEG experiment and analysis were performed in a group of 6 male rats.

**Statistical Analysis:** Sample size was calculated by using GPower [24], which suggested a minimum group size of 4. Initial group size was set to 8 in order to allow for loss of animals as they aged. In this study the threshold for statistical significance was set to 0.05. The statistical test used to analyze each data set is described in the appropriate figure legends. If three or more comparisons were required within a group of animals a repeated measures analysis of variance (RM-ANOVA) with Tukey’s multiple comparisons post-hoc test was employed. Sphericity was corrected with Geisser-Greenhouse and normality was evaluated visually. When only 2 conditions were assessed, either a paired or an unpaired T-test was employed. Data was analyzed and graphed using GraphPad Prism 10 software. Data were expressed and plotted graphically as mean ± standard deviation (SD). The experiments shown in this manuscript were done in an unblinded manner, although experimental order and drug application were randomized. All data obtained in this study are shown in this manuscript.

## Results

### Supplementing Dex with Mg^2+^ suppressed responses to Noxious stimuli in all age groups and in both sexes

We evaluated whether supplementing Dex with Mg^2+^ created an effective anesthetic. Dex supplemented with Mg^2+^ was compared to Dex alone to see if it could suppress responses to noxious stimuli. Anesthetic efficacy was assessed by clamping the rat tail every 15 minutes during the sixty-minute infusion. Table 1 shows that supplementing Dex with Mg^2+^ suppressed responsiveness to the tail clamp, while Dex alone did not. Young female rats receiving Dex with Mg^2+^ had significantly fewer positive responses (4 out of 32) compared to those receiving Dex alone (28 out of 32; p < 0.0001, Fisher’s exact test). Higher Mg^2+^ levels (100 mg/kg bolus, 50 mg/kg/hr infusion) eliminated all responses to the painful tail clamp.

**Table 1:** Testing Dex alone or Dex supplemented with Mg^2+^ in female rats that were either 3-4 months old (left 2 columns) or 11-12 months old (right 2 columns). A noxious stimulus was applied at different time points in each experiment, as indicated. Responses were tabulated and are presented numerically in the table. Statistical difference is calculated with Fisher’s exact test. Repeating the tail clamp experiment in the two young rats that responded to the stimulus with same dose of Dex but an increased level of Mg^2+^ (100 mg/kg bolus and 50 mg/kg/hr infusion), completely suppressed all response to the stimulus. None of the older rats responded to the tail clamp at the lower dose of Mg^2+^ with Dex.

	Young Female Rats (3–4 months)		Older Female Rats (11–12 months)	
Time of test	Dex 10 µg/ kg bolus, then 12 µg/kg/hr infusion	Dex 10 µg/kg & Mg^2 +^ 75 mg/kg bolus, then Dex 12 µg/kg/hr & 37.5 mg/kg/hr infusion	Dex 10 µg/ kg bolus, then 12 µg/kg/hr infusion	Dex 10 µg/ kg & Mg^2 +^ 50 mg/kg bolus, then Dex 12 µg/kg/hr & 25 mg/kg/hr infusion
15 min	5 responses/8 tests	0 response/8 tests	3 responses/8 tests	0 response/8 tests
30 min	7/8	0/8 tests	7/8	0/8
45 min	8/8	2/8	7/8	0/8
60 min	8/8	2/8	8/8	0/8
Total	28/32	4/32	25/32	0/32
Statistics on total Fisher’s exact test		P < 0.0001 compared to Dex alone		P < 0.0001 compared to Dex alone

Dex and Mg^2+^ completely suppressed all responses to the noxious stimulus (see Table 1), when tested in older female rats (11–12 months), significantly different than Dex alone (P < 0.0001). In the older rats, a dose of Mg^2+^ lower than that used in the young female rats suppressed all response to the noxious stimulus.

Young male rats, 3–4 months old, responded to the tail clamp in 31 out of 32 tests, when they were infused with Dex alone (10 μg/kg bolus, then a 12 μg/kg/hr infusion for 60 minutes, Table 2). These rats responded 4 times in 32 tests when they received Dex augmented with Mg^2+^ (100 mg/kg bolus, then infusion 50 mg/kg/hr for 60 minutes) which was significantly different than Dex alone (p < 0.0001: Fisher’s exact test, Table 2). Adding a very low dose of Midazolam (0.3 mg/kg bolus, then infusion 0.1 mg/kg/hr infusion) in addition to the Mg^2+^, prevented any of the Dex/ Mg^2+^ rats from responding to the tail clamp (0 responses in 32 tests, p < 0.0001 compared to Dex alone; Table 2).

**Table 2:** Testing Dex alone or Dex supplemented with Mg^2+^ or Dex supplemented with both Mg^2+^ and midazolam in 3–4-month-old male rats. A noxious stimulus was applied at different time points in an experiment. Responses were tabulated and are presented numerically in the table. Statistical difference is calculated with Fisher’s exact test. Data were obtained from 8 male rats. Note that Dex with Midazolam at 0.3 mg/kg bolus and 0.1 mg/kg/hr was not sufficient to suppress the tail clamp tests in these rats.

Young Male Rats (3–4 months)			
Time of Test	Dex 10 µg/ kg bolus, then 12 µg/kg/hr infusion	Dex 10 µg/kg & Mg^2 +^ 100 mg/kg bolus, then Dex 12 µg/kg/hr & Mg^2 +^ 50 mg/kg/hr infusion	Dex 10 µg/kg & Mg^2 +^ 100 mg/kg & Midazolam 0.3 mg/kg bolus, then Dex 12 µg/kg/hr & Mg^2 + ^50 mg/kg/hr & Midazolam 0.1 mg/kg/hr infusion
15 min	7 responses/8 tests	0 responses/8 tests	0 responses/8 tests
30 min	8/8	0/8	0/8
45 min	8/8	1/8	0/8
60 min	8/8	3/8	0/8
Total responses	31/32	4/32	0/32
Statistics on total Fisher’s exact test		P < 0.0001 compared to Dex alone	P < 0.0001 compared to Dex alone

In older male rats (11–12 months of age), supplementing Dex with Mg^2+^ alone completely suppressed responses to the noxious stimulus (Table 3). There was a small increase in heart rate at the late time points.

**Table 3:** Testing Dex supplemented with Mg^2+^ in 11-12 month old male rats. A noxious stimulus was applied at the times indicated. Responses were tabulated and are presented numerically in the table. Statistical difference is calculated with Fisher’s exact test. This same group of rats gained a significant amount of weight from 3–4-month-old to 11-12-month-old, p = 0.0014.

Older Male Rats (11–12 months)	
Time of test	**Dex (10 µg/kg) & Mg**^**2+**^ **(100 mg/kg) bolus, then Dex (12 µg/kg/hr) & Mg**^**2 +**^** 50 mg/kg/hr infusion**
15 min	0 responses/8 tests
30 min	0/8
45 min	0/8
60 min	0/8
Total	0/32

Dex supplemented with Mg^2+^ completely suppressed all responses to the noxious stimulus in both old female (20–21 months) and male rats (18–19 months) (Table 4).

**Table 4:** Testing Dex alone or Dex supplemented with Mg^2+^ in old female (20-21 months) and male rats (18-19 months). The rats were exposed to Dex with Mg^2+^ as indicated. A noxious stimulus was applied at different time points in an experiment. Responses were tabulated and are presented numerically in the table. The data is shown for illustrative purposes only.

	Old Female Rats (20–21 months, n = 6)	Old Male Rats (18–19-months, n = 4)
Time of Test	**Dex 10 µg/kg & Mg** ^**2 +**^ ** 100 mg/kg bolus, then Dex 12 µg/kg/hr & Mg** ^**2 +**^ ** 50 mg/kg/hr infusion**	**Dex 10 µg/ kg & Mg** ^**2 +**^ ** 100 mg/kg bolus, then Dex12 µg/kg/hr & Mg** ^**2 +**^ ** 50 mg/kg/hr infusion**
15 min	0 responses/6 tests	0 responses/4 tests
30 min	0/6	0/4
45 min	0/6	0/4
60 min	0/6	0/4
Total responses	0/24	0/16

Dex supplemented with Mg^2+^ effectively suppressed responses to the tail clamp stimulus in both sexes, at all ages.

### Supplementing Dex with Midazolam suppressed responses to Noxious stimuli at all ages in rats of both sexes

Table 5 shows data, left two columns, from young female rats, 3–4 months old, that received a bolus of Dex (10 µg/ kg) alone over 5 minutes, followed by a 60-minute infusion of Dex (12 μg/kg/hr). In a different experiment, these same rats received the identical Dex bolus (10 μg/kg) while also receiving a bolus of midazolam (0.6 mg/kg) followed by a 60-minute infusion of Dex (12 μg/kg/hr) and midazolam (0.3 mg/kg/hr).

**Table 5:** Testing Dex alone or Dex supplemented with midazolam in 3–4-month-old (left two columns) and 11–12-month-old female rats (right two columns). A noxious stimulus was applied every 15 minutes during an experiment. Responses were tabulated and statistical difference is calculated with Fisher’s exact test. Data were obtained from the same group of 8 female rats.

	Young Female Rats (3–4 months)		Older Female Rats (11–12 months)	
Time of test	Dex 10 µg/ kg bolus, then 12 µg/kg/hr infusion	Dex 10 µg/kg & Midazolam 0.6 mg/kg bolus, then Dex 12 µg/kg/hr & 0.3 mg/kg/hr infusion	Dex 10 µg/kg bolus, then 12 µg/kg/hr infusion	Dex 10 µg/kg & Midazolam 0.6 mg/kg bolus, then Dex 12 µg/kg/hr & 0.3 mg/kg/hr infusion
15 min	5 responses/8 tests	0 response/8 tests	3 responses/8 tests	0 response/8 tests
30 min	7/8	0/8 tests	7/8	0/8
45 min	7/8	0/8	7/8	0/8
60 min	8/8	0/8	8/8	0/8
Total	27/32	0/32	25/32	0/32
Statistics on total Fisher’s exact test		P < 0.0001 compared to Dex alone		P < 0.0001 compared to Dex alone

The rats had their tails clamped at four different times (15, 30, 45 and 60 during infusion) after drug infusions started. Aggregating the data from 4 time points, there were no responses to the tail clamp when animals received Dex supplemented with midazolam (0 responses out of 32 tests), which was significantly different than the 27 positive responses out of 32 total tests when those same animals received Dex alone (27 responses out of 32 tests; p < 0.0001, Fisher’s exact test).

In female rats 11–12 months old, supplementing Dex with midazolam completely suppressed all responses to the tail clamp (0 responses out of 32 tests; p < 0.0001 when compared to Dex alone, Table 5, right two columns).

Young male rats (3–4) responded to the noxious stimulus when they received Dex alone. In contrast, when the Dex was supplemented with midazolam (0.3 mg bolus followed by 0.1 mg/kg/hr infusion the rats responded 9 out of 32 times to the tail clamp, which was significantly different than Dex alone (p < 0.05). Increasing midazolam levels (0.6 mg bolus followed by 0.3 mg/kg/hr infusion) no rat responded to the noxious stimulus (0 responses in 32 tests, p < 0.0001 compared to Dex alone) (Table 6).

**Table 6:** Testing Dex alone and Dex with different doses of Midazolam in male rats that were 3-4 months old. The stimulus was applied at different time points in an experiment. Responses were tabulated and are presented numerically in the table. Statistical difference is calculated with Fisher’s exact test. Data were obtained from 8 male rats.

Young Male Rats (3–4 months)			
Time of test	**Dex bolus 10 µg/ kg, then 12 µg/kg/hr infusion**	**Dex 10 µg/kg & Midazolam 0.6 mg/kg bolus, then Dex 12 µg/kg/hr & 0.3 mg/kg/hr infusion**	**Dex 10 µg/kg & Midazolam 0.3 mg/kg bolus, then Dex 12 µg/kg/hr & 0.1 mg/kg/hr infusion**
15 min	7 responses/8 tests	0 responses/8 tests	0 responses/8 tests
30 min	8/8	0/8	1/8
45 min	8/8	0/8	4/8
60 min	8/8	0/8	4/8
Total	31/32	0/32	9/32
Statistics on total Fisher’s exact test		P < 0.0001 compared to Dex alone	P < 0.05 compared to Dex alone

### Vital signs

Fig 1A shows that supplementing Dex with either Mg^2+^ or midazolam did not alter heart rate relative to Dex alone, in female rats that were 11–12 months. Using a repeated measures ANOVA we found no difference among the groups (Dex alone, Dex with Mg^2+^, Dex with midazolam). Similarly, Fig 1B shows that there was no change in blood pressure in this same cohort of rats when comparing Dex alone to Dex supplemented with Mg^2+^ or Dex supplemented with midazolam. The data suggest that cardiovascular changes appear to result from Dex administration alone.

No differences in vital signs were observed in male rats 11–12 months old when comparing Dex alone to Dex supplemented with either Mg^2+^ or midazolam. (Fig 2). In general, there was significant variability in HR and BP between rats in a group and female rats had lower HR and BP as compared to male rats. That made it important for rats to serve as their own controls.

To test the safety and efficiency of Dex and Mg^2+^ in aged rats, we evaluated six female and four male rats, 20–21 months old and 18–19 months old, respectively (Fig 3). Dex and Mg^2+^ produced similar HR and BP changes in the old female and male rats as they did in the younger rats. MBP was not altered by Dex with Mg^2+^ in any of the rats while HR was lowered by Dex with Mg^2+^.

### A reversal cocktail enhanced emergence from Dex sedation/anesthesia

Recovery from Dex sedation is typically extremely slow. In a previous study we demonstrated that a reversal cocktail comprised of low dose atipamezole with caffeine reduced emergence times from Dex sedation by >95% [24].The reversal agent reversed Dex supplemented with either propofol or sevoflurane [13]. In the current study, animals were injected with either saline (control) or with atipamezole (10 µg/ kg) and caffeine (25 mg/ kg). The rats were then placed on their backs inside a cage. The time to emerge from anesthesia/ sedation was defined as the time required for the rats to flip over and stand with 4 paws on the bottom of the cage (recovery of righting reflex – RORR time). In our study, RORR is used as a proxy for recovery of consciousness similar to our previous studies (see ref 20).

Atipamezole and caffeine reversed Dex supplemented with Mg^2+^ or Dex supplemented with midazolam. In all cases, emergence was accelerated by >95%, significantly different than emergence without the aid of atipamezole and caffeine in female rats (Fig 4) and in male rats (Fig 5) 11–12 months of age. Furthermore, the rats were alert and active when they recovered their righting reflex after atipamezole and caffeine. More specific and validated tests will be needed to confirm our impression that the rats completely recovered their cognitive abilities.

### EEG analysis: Dex with Mg^2+^ vs Dex with Midazolam

Dex is sedative but not amnestic when used alone [26]. Mg^2+^, a constituent ion in the CSF, is also not amnestic. Midazolam, by contrast, is strongly amnestic even at low doses [27]. We recorded EEG activity during infusions of Dex and midazolam, which make recall, and awareness very unlikely, to serve as a baseline for comparison to Dex infusion with Mg^2+^. Fig 6 plots 10-minute spectrograms from female and male rats (12 months old) anesthetized with Dex and Mg^2+^ (Figs 6A, 6C) and Dex and midazolam (Figs 6B, 6D). The spectrograms are similar across conditions and genders. All four spectrograms were obtained near the midway point of the continuous infusion. Note that the same female rat was used to make both traces in panels A/B and the same male rat was used for both traces in panels C/D.

Fig 7A displays power spectra for 11–12-month-old female rats comparing Dex and Mg^2+^ [blue trace] versus Dex and midazolam [red trace]. Delta (0.5–4 Hz), Theta (4–8 Hz), and Alpha (8–12 Hz) bands represented >95% of power and were not different between conditions. The Beta band (15–25 Hz) was significantly higher for Dex and midazolam than Dex and Mg^2+^ (p = 0.001). Fig 7B plots power spectra for male rats. Delta, Theta, and Alpha bands constituted >95% of power and were not different. Theta band was significantly higher with Dex with Mg^2+^ (p < 0.05). The depth of anesthesia based on EEG was similar between Dex with Mg^2+^ vs Dex with midazolam. An unpaired T-test was used to determine differences in frequency bands.

### Abdominal surgery in older female rats

A successful anesthetic must produce immobility during surgery for it to be useful [26]. Abdominal surgery was carried out in one group of 8 female rats (11–12 months) (see Fig 8). Four rats were anesthetized with Dex supplemented with Mg^2+^. Four rats were anesthetized with Dex and midazolam. The abdominal incision from one rat is shown in Fig 8A. Figs 8B and 8C show heart rate, SpO_2_ and mean arterial pressure immediately before and after skin incision for Dex and Mg^2+^ and Dex and Midazolam anesthesia. In all 8 rats, surgery did not produce motor or autonomic responses during the entire procedure. The results suggest that Dex supplemented with Mg^2+^ produces effective anesthesia with deep levels of unconsciousness, immobility and antinociception with similar efficacy to Dex with Midazolam.

No adverse events were observed in these experimental sessions.

## Discussion

### Anesthetics in General Use are not as Innocuous as previously thought

General anesthetics are meant to be reversible and nontoxic, but evidence suggests they may not be as benign as once thought, especially for the elderly, who are the largest consumers of procedural care [28]. Older adults worry about brain health and preserving neurocognitive function after surgery [29]. Postoperative cognitive dysfunction (POCD) is associated with deficits in executive function and memory [14,30–32]. There is growing interest in the link between anesthetic exposure and dementia including Alzheimer’s disease [33]. Postoperative delirium, common in the elderly, is linked to longer hospital stays, higher care costs, increased institutionalization, morbidity, mortality, and higher readmission rates [34,35]. Patients with delirium often experience functional decline and decreased independence in daily living [36]. Delirium is a predictor of cognitive impairment after surgery [14,36–39]. While cognitive changes are usually short-lived, they can persist in some patients, leading to memory loss, impaired cognitive function, psychomotor issues, and depression. POCD is associated with early retirement, increased dependence on social services, and higher mortality [40]. After cardiac surgery, cognitive problems can affect up to 42% of patients five years later [41].

Intravenous anesthetics like propofol and etomidate potentiate GABA_A_ receptor currents thereby suppressing neuronal activity [7]. Potentiation of GABA_A_ receptor activity is also foundational to the mechanism of inhalational anesthetics like sevoflurane. Our previous study showed that the α_2_ agonist Dex when combined with low dose propofol produced robust general anesthesia sufficient to perform surgery in rats that could be rapidly reversed by a reversal cocktail composed of low dose atipamezole and caffeine [13].

### Dex supplemented with Mg^2+^ May create a safer anesthetic

In the current study, Dex supplemented with Mg^2+^ created a mechanistically unique anesthetic, although it is possible that Mg^2+^ can modulate GABA_A_ receptor activity [42]. Dex, an α_2_ agonist, lowers [cAMP]_i_ and inhibits neuronal activity, while elevating extracellular Mg^2+^ in the brain inhibits voltage-gated Ca^2+^ channels as well as ligand-gated channels permeable to Ca^2+^ like NMDA receptors [43]. Dex appears to reduce information transfer in local and global brain networks in humans [44] and engender a functional disconnect between brain regions the likely mechanism leading to unconsciousness [45,46]. Dex increases slow-delta oscillations while increasing frontal spindle oscillations. Dex EEGs closely approximate those seen during human non-rapid eye movement (NREM) sleep [47,48] and may represent a less stressful form of unconsciousness. Dex is recognized as neuroprotective agent [49–51] as is Mg^2+^ [16–18]. Dex supplemented with Mg^2+^ is mechanistically different than current anesthetics and may represent a safer, more neuroprotective alternative.

Supplementing Dex with midazolam created a powerful anesthetic. Dex supplemented with Mg^2+^ was compared to Dex supplemented with midazolam. Both drug combinations suppressed almost all responses to noxious tail clamping. Both drug combinations suppressed motor and autonomic responses during abdominal surgery. Midazolam is known to be strongly amnestic even at low concentrations. Dex/Mg^2+^ produced EEG spectra similar to Dex/midazolam, suppressing high frequency components known to be associated with memory, suggesting that the Dex/Mg^2+^ combination may be amnestic. Greater than 95% of the frequencies present in EEG recordings were less than 15 Hz in both Dex/Mg^2+^ and Dex/Midazolam. Furthermore, both drug combinations produced less burst suppression than anesthetics like sevoflurane when used by itself near its EC_50_ [13]. Studies have shown that that burst suppression may be associated with increased delirium [52] suggesting that Dex/ Mg^2+^ may result in less delirium.

### Dex supplemented with Mg^2+^ was effective in both male and female rats at all ages

We observed differences in sensitivity to Dex/ Mg^2+^ between female and male rats and at different age groups. Both female and male rats 3–4 months of age required the highest doses of Mg^2+^ to effectively supplement Dex. Even with a high dose of Mg^2+^, three young male rats responded to the tail clamp. In subsequent sessions, total inhibition of all reactions to the noxious stimulus was achieved in these same rats when a very low dose of midazolam was added without increasing the dose of Mg^2+^ (Table 2). Dex with Mg^2+^ was most effective in female rats 11–12 months of age, where a lower dose of Mg^2+^ completely suppressed all response to the noxious stimulus (Table 1). Two factors may contribute to these differences. Firstly, Mg^2+^ is almost entirely cleared by the kidney. Glomerular filtration rates (GFR) are different between males and females and decrease with age [53,54]. Secondly, Mg^2+^ was administered based on weight and weight changes were significant from the age of 3–4 months to 11–12 months. A recent report also showed sensitivity to dexmedetomidine appeared different in male and female rats [55]. Clinically, pain meds routinely supplement anesthetics. Otherwise, very high dosages of anesthetics are required to suppress pain. If Dex/ Mg^2+^ is used clinically, the addition of pain medications would suppress rare responses to noxious stimuli, while preserving their neuroprotective potential.

### Does Dex supplemented with Mg^2+^ produce an effective anesthetic suitable for surgery?

Rats anesthetized with Dex supplemented by Mg^2+^ showed no response to surgery, but they did respond a few times to the tail clamp. Why? Surely the tail clamp did not produce a more powerful stimulus than the painful abdominal surgery. One flaw in our study is that we were not able to measure drug concentrations in the anesthetized rats. We hypothesize that the Mg^2+^ levels were diminishing with time during the tail clamp procedure even with the continuous infusion. There were no responses to surgery as the entire surgical procedure took place within 30 minutes after Dex and Mg^2+^ were first introduced, leaving less time for the drug levels to decrease. Similarly, we saw no responses to the tail clamp at early times, only at later times after the infusion process had been ongoing. It is quite possible that the continuous infusion that we employed did not keep up with Mg^2+^ excretion by the kidneys. What does this result mean for surgeries that can last for hours? Probably very little. Even at later times Dex with Mg^2+^ is more effective than either sevoflurane or propofol, popular anesthetics, when they are used alone at the dosages employed for surgeries (near MAC levels). To make sevoflurane or propofol effective anesthetics pain management strategies are employed, typically opiates. This same strategy will create an extremely effective anesthetic using Dex/ Mg^2+^. Alternatively, Mg^2+^ infusion can be more closely matched with that of excretion.

### Dex dosing

Our anesthetic combinations emphasize Dex as the central component of a general anesthetic agent and employs a minimal dose of a second agent in an adjunctive capacity. This dose of Dex was sufficient to cause LORR, but not able to block responses to tail clamp tests in rats [13,20]. Dex doses up to 3 µg/kg/hr are commonly used for pediatric procedural sedation, such as for MRI scans [56]. In contrast, Dex 0.5–0.75 mg/kg IP is used in combination with ketamine at 75–150 mg/kg IP to anesthetize small animals, like rats, for surgery (see for example ref [57]).

### Dex supplemented with Mg^2+^ produces an anesthetic that is rapidly reversible

There is currently no FDA approved reversal available for any of the commonly employed anesthetics clinically. The process of anesthesia emergence is passive. As we gain a better understanding of the mechanisms of emergence from anesthesia, there are significant efforts to search for clinically effective and safe methods for reversal. The goal is to improve patient care, particularly in vulnerable populations. A recent review, by a panel of experts, has summarized developments in the field [58].

A combination of low dose atipamezole and caffeine completely reverses Dex sedation [24]. Emergence from unconsciousness produced by Dex supplemented with either Mg^2^+ or midazolam was reversed with equal efficacy by the reversal cocktail. Reversibility is critical because emergence from Dex anesthesia is very slow.

A recent study showed that a large dose of amphetamine, a potent stimulant with a half-life of ~10 hours, could rapidly reverse Dex by itself [55]. A combination of low dose atipamezole with caffeine may be able to reverse dexmedetomidine rapidly while minimizing the prolonged effects of the stimulant. The half-life of atipamezole is almost identical to that of Dex – they would disappear in lock step, leaving caffeine in isolation. The half-life of caffeine is ~ half that of amphetamine. Releasing patients with caffeine, as opposed to Schedule II amphetamine in their systems, may be the preferred option.

Finally, we tested a limited number of aging rats. Rats of both sexes near 20 months of age maintained stable vital signs during Dex and Mg^2+^ infusion. Further studies will be required to make definitive statements about anesthesia by Dex/Mg^2+^ and emergence in old rats since the group sizes were so limited.

### Limitations

Plasma concentrations of drugs were not measured (including Dex, Mg^2+^ and midazolam) and may have been changing through the experiments. There were no baseline EEG measurements available as the EEG leads were placed after the rats were anesthetized. Although the drugs appeared effective for surgeries, these surgeries were completed in the first 30 minutes after a bolus of both drugs. Longer surgeries were not tested. Whether Dex/Mg^2+^ makes an anesthetic that reduces the need for opioids intraoperatively in human patients and minimizes side effects postoperatively remains undetermined. Whether Dex/ Mg^2+^ reduces neuroapoptosis in neonatal animals and prevents cognitive decline or emergence delirium in the elderly remains undetermined. Although both Dex and Mg^2+^ exhibit neuroprotective properties, whether they do so when combined remain to be confirmed. Atipamezole is not yet approved in human use due to the high dose requirement. The strategy to reverse Dex/ Mg^2+^ with low dose atipamezole and caffeine awaits confirmation in human trials. A larger cohort of elderly rats will be needed to confirm the efficacy of the Dex/ Mg^2+^ combination in aged rats and the ability of caffeine and atipamezole to reverse the drugs. Validated psychological testing in old rats will be required to determine whether they completely recover their cognitive abilities after rapid emergence from anesthesia.

In summary, our data suggests that Dex augmented with Mg^2+^ creates a useful anesthetic, with a favorable safety profile with neuroprotective effects and is completely reversible. The potential next step is to translate Dex/ Mg^2+^ to the human population after a safe and effective reversal is successfully tested.

## Supporting information

S1 Fig EEG electrode placement. (PDF)

S2 Fig Animal usage. (PDF)
