# Supplementary figures and images for: Studies in rats of a target specific and reversible general anesthetic with a favorable safety profile
Source: PLoS One. 2025 Nov 4;20(11):e0335589. doi: 10.1371/journal.pone.0335589 (PMC12585021; doi:10.1371/journal.pone.0335589)

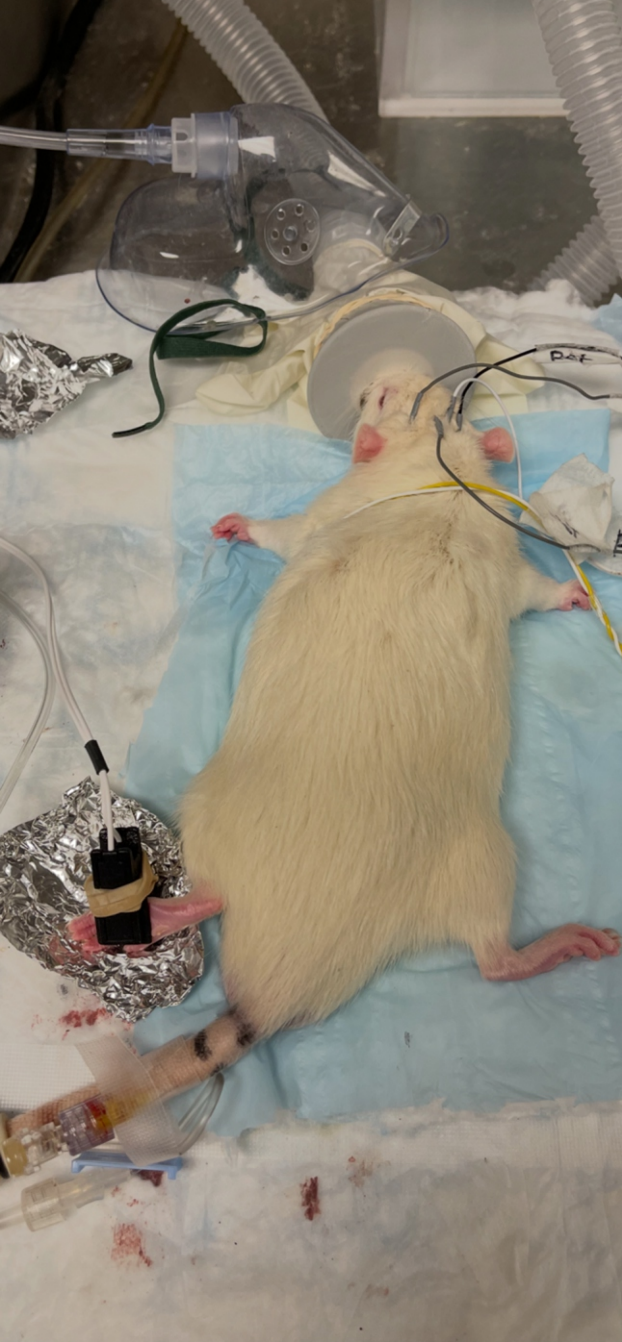

Supplement: S1 Fig — (PDF) [file pone.0335589.s001.pdf]
