# Supplementary material for: Studies in rats of a target specific and reversible general anesthetic with a favorable safety profile
Source: PLoS One. 2025 Nov 4;20(11):e0335589. doi: 10.1371/journal.pone.0335589 (PMC12585021; doi:10.1371/journal.pone.0335589)
Supplement: S2 Fig — (PDF) [file pone.0335589.s002.pdf]

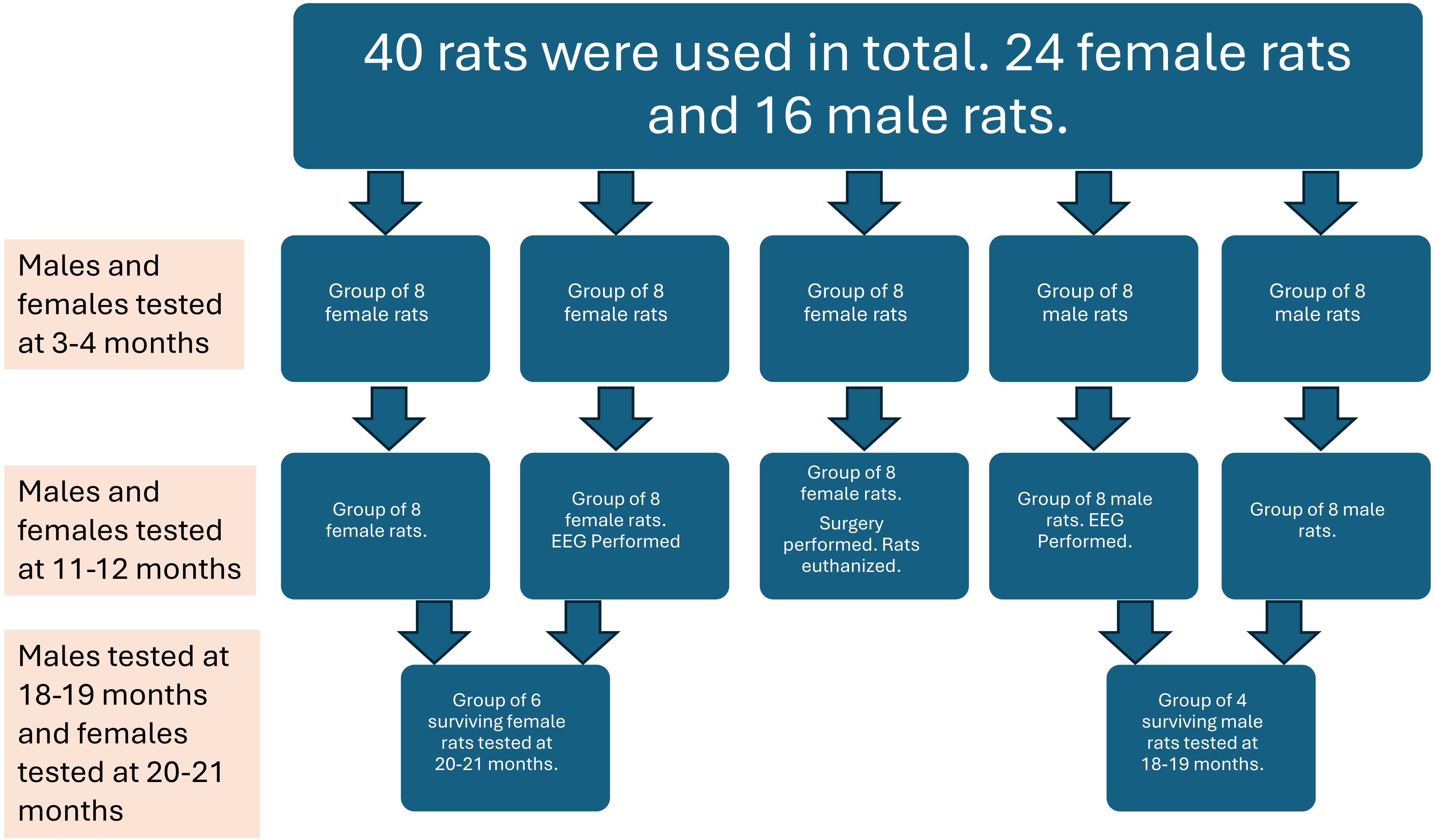
40 rats were used in total. 24 female rats and 16 male rats.
Males and females tested at 3-4 months
Group of 8 female rats
Group of 8 female rats
Group of 8 female rats
Group of 8 male rats
Group of 8 male rats
Males and females tested at 11-12 months
Group of 8 female rats.
Group of 8 female rats. EEG Performed
Group of 8 female rats. Surgery performed. Rats euthanized.
Group of 8 male rats. EEG Performed.
Group of 8 male rats.
Males tested at 18-19 months and females tested at 20-21 months
Group of 6 surviving female rats tested at 20-21 months.
Group of 4 surviving male rats tested at 18-19 months.
